# Identification of Mammalian Protein Quality Control Factors by High-Throughput Cellular Imaging

**DOI:** 10.1371/journal.pone.0031684

**Published:** 2012-02-20

**Authors:** Gianluca Pegoraro, Ty C. Voss, Scott E. Martin, Pinar Tuzmen, Rajarshi Guha, Tom Misteli

**Affiliations:** 1 Cell Biology of Genomes, National Cancer Institute, National Institutes of Health, Bethesda, Maryland, United States of America; 2 NCI High Throughput Imaging Facility, National Cancer Institute, National Institutes of Health, Bethesda, Maryland, United States of America; 3 National Institutes of Health Center for Translational Therapeutics, Rockville, Maryland, United States of America; University of Pittsburgh, United States of America

## Abstract

Protein Quality Control (PQC) pathways are essential to maintain the equilibrium between protein folding and the clearance of misfolded proteins. In order to discover novel human PQC factors, we developed a high-content, high-throughput cell-based assay to assess PQC activity. The assay is based on a fluorescently tagged, temperature sensitive PQC substrate and measures its degradation relative to a temperature insensitive internal control. In a targeted screen of 1591 siRNA genes involved in the Ubiquitin-Proteasome System (UPS) we identified 25 of the 33 genes encoding for 26S proteasome subunits and discovered several novel PQC factors. An unbiased genome-wide siRNA screen revealed the protein translation machinery, and in particular the EIF3 translation initiation complex, as a novel key modulator of misfolded protein stability. These results represent a comprehensive unbiased survey of human PQC components and establish an experimental tool for the discovery of genes that are required for the degradation of misfolded proteins under conditions of proteotoxic stress.

## Introduction

Cellular proteins must assume and maintain their native 3D conformation in order to be functionally active. Partial folding or misfolding renders proteins non-functional, and improperly folded proteins may become toxic to the cell [1], [2]. Accurate folding of proteins is particularly critical to prevent the formation of cellular aggregates and is implicated in human disease. Misfolded proteins tend to expose highly hydrophobic surfaces that are normally buried in their interior. Given the hydrophilic nature of the cellular medium, hydrophobic surfaces from different misfolded proteins tend to interact with each other and to form cellular aggregates [1]. Protein misfolding can lead to the disruption of protein homeostasis in a dominant negative fashion and may ultimately cause cell death as seen in Parkinson, Alzheimer and Huntington disease [3], [4].

Cells possess dedicated Protein Quality Control (PQC) pathways to ensure maintenance of the proteostatic equilibrium [3]. One arm of the PQC systems consists of protein chaperones that bind to unfolded proteins, including newly synthesized proteins, and, by hydrolysing ATP, actively aid in attaining mature protein conformation [1], [5]. The PQC system also acts on mature, properly folded but metastable proteins that have a tendency to revert to a non-native state, particularly in conditions of proteotoxic stress such as in the presence of oxidizing agents or elevated temperature [5]. A second arm of the PQC system clears proteins damaged beyond repair [6]. This pathway includes E1-, E2- and E3-ubiquitin ligases, which are recruited by the chaperones themselves and poly-ubiquitinate irreversibly misfolded proteins, thus targeting them for proteolysis by the 26S proteasome. Finally, a multitude of dedicated transcription factors responds to proteotoxic stimuli by up-regulating the transcription of genes that promote PQC [7], [8].

The PQC pathways are spatially compartmentalized according to the subcellular location of their misfolded substrates. Specific pathways dealing with misfolded proteins exist in the cytoplasm, in the ER and in mitochondria [8]–[11]. Previous work in S. *cerevisiae* also suggests that the nucleus contains E3-ubiquitin ligases dedicated to PQC [12]–[14]. In support, there is evidence that some misfolded substrates are also degraded in the mammalian nucleus [15], [16].

Alterations to protein homeostasis, either due to an increase in the load of misfolded proteins or due to a failure of the PQC systems to respond to proteotoxic stress, underlie common human neurological diseases, aging and cancer [5], [17]. Hence, the discovery and characterization of novel genes that belong either to the PQC pathways, or that regulate PQC activity upon external cues, is of great basic and applied importance. Expression of pathologically misfolded proteins in small model organisms such as S. *cerevisiae*, C. *elegans* and *D. melanogaster* has been used in reverse genetic screens to identify cellular modifiers of protein misfolding and aggregation [18]–[21]. However, no cellular assays for the systematic and unbiased discovery, for example by RNAi screening, of PQC factors in mammalian cells have been reported. Given the lack of tools to study PQC systems in mammalian cells in an unbiased fashion, we designed and implemented a cell-based assay to measure the degradation of a misfolded protein in intact cells. Here we describe a quantitative, high-content fluorescence microscopy PQC assay amenable to high-throughput screening and we use it in a targeted siRNA screen of 1591 genes belonging to the Ubiquitin Proteasome Systems (UPS) and in a genome-wide unbiased global screen. We identify several novel players in the human PQC pathway.

## Results

### Assay Design

**Figure 1:**
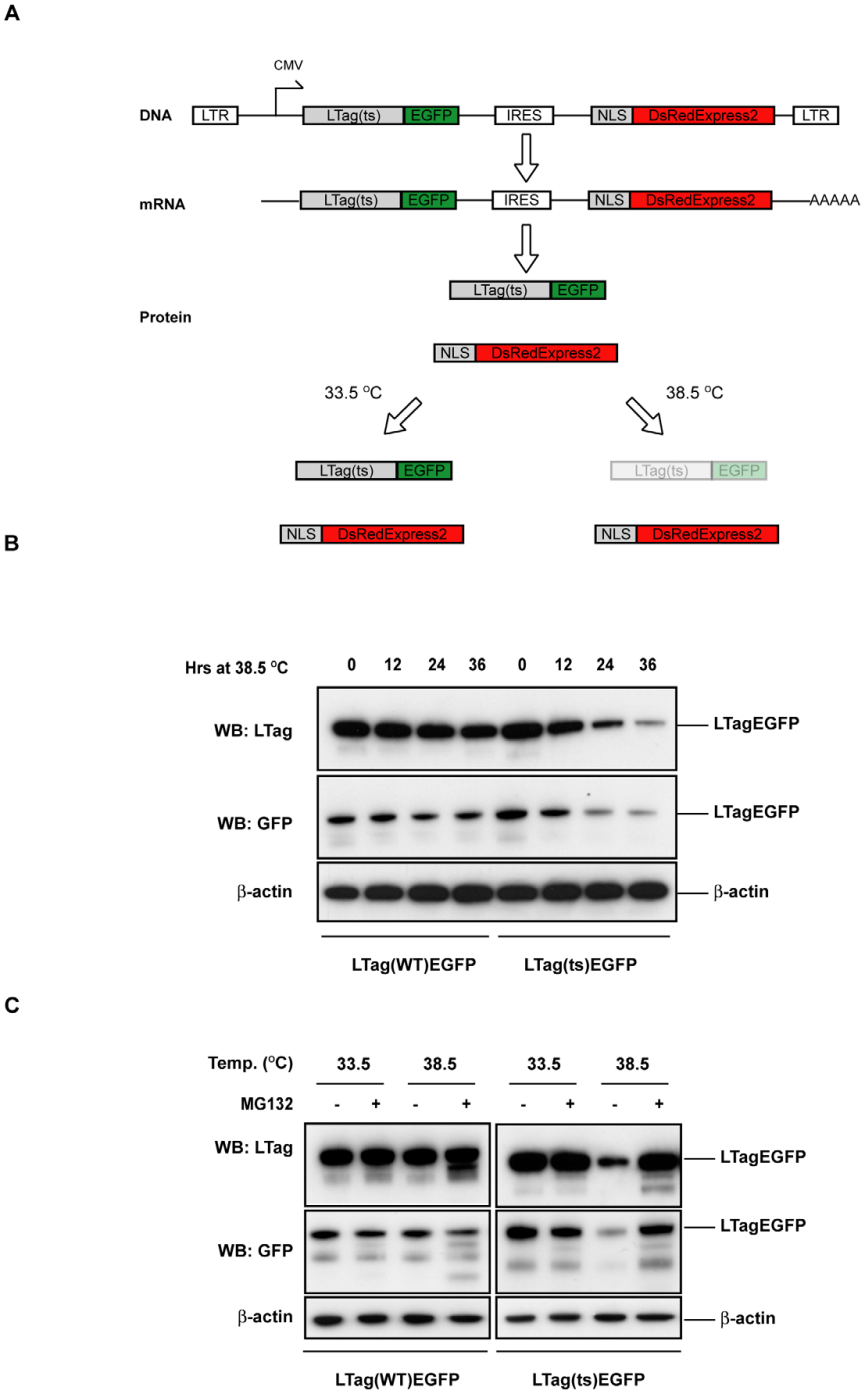
A temperature sensitive (ts) allele of the SV40 Large T antigen (LTag) fused to EGFP engages the PQC machinery. a) Outline of the fluorescent PQC reporter system in mammalian cells. LTR: Retroviral Long Terminal Repeat, CMV: immediate early cytomegalovirus promoter, LTag; SV40 Large T antigen, ts: temperature sensitive, IRES: internal ribosome entry site, NLS: nuclear localization signal. b) U2OS cells stably expressing either a wild-type (WT) or a temperature-sensitive (ts) allele of LTag fused to EGFP were grown at 33.5°C and then shifted to 38.5°C for the indicated amount of time. Total cell lysates were probed in Western Blotting with the indicated antibodies. c) Same as b), except that cells were either treated with DMSO or the proteasome inhibitor MG132 at a final concentration of 2 µM.

We set out to identify novel human genes involved in PQC in the cell nucleus in an unbiased fashion using a cell-based assay. In order to measure the activity of the PQC pathways in the cell nucleus in living cells, we took advantage of a temperature-sensitive (ts) allele of the nuclear localized SV40 virus Large T antigen (LTag(ts)) [22]. SV40-LTag(ts) is stable at the permissive temperature of 33.5°C but becomes unstable and is rapidly degraded at the non-permissive temperature of 38.5°C (Figure S1A). We chose SV40-LTag because it represents a class of long-lived nuclear proteins representative of many chromatin associated proteins, transcription factors and nuclear lamins whose degradation is only poorly characterized. In order to use the LTag(ts) PQC probe in high-throughput, quantitative fluorescence microscopy experiments we created a stable cell line containing a recombinant LTag(ts) protein fused in frame to EGFP (Figure 1A). In addition, we placed a red fluorescent protein fused to a nuclear localization signal (NLS-DsRedExpress2) downstream of an internal ribosome entry site (IRES) (Figure 1A). This arrangement guarantees that both open reading frames encoding the fluorescent proteins are co-cistronically transcribed and translated at a constant ratio. Degradation of LTag(ts)-EGFP can be measured as a change in the ratio between nuclear green and red fluorescence signals (Figure 1A). The generation of LTag(ts)-EGFP and NLS-DsRedExpress2 from the same RNA rules out changes due to transcriptional or translational effects and the presence of the NLS-DsRedExpress2 protein allows internal normalization of sample-to-sample fluorescence intensity fluctuations in high-throughput screening formats [23].

As expected, steady-state levels of LTag(ts)-EGFP stably expressed in U2OS cells were stable at the permissive temperature (33.5°C), but declined 24 hrs after the switch from the permissive to the restrictive temperature as determined by Western Blotting (38.5°C, Figure 1B). Degradation was specific to the temperature sensitive LTag mutant, since LTag(WT)-EGFP levels remained unchanged, thus ruling out possible transcriptional or translational effects due to the elevated temperature (Figure 1B). Furthermore, treatment with the proteasome inhibitor MG132 completely blocked the reduction of LTag(ts)-EGFP at the restrictive temperature (Figure 1C). The degradation properties of the LTag(ts) fusion protein were not due to the presence of the EGFP tag, since the kinetics of degradation of the EGFP-tagged version of LTag(ts) and LTag(WT) and the sensitivity of LTag(ts)-EGFP degradation were identical to their untagged counterparts (Figure S1A and S1B). These results indicate that at the restrictive temperature LTag(ts)-EGFP, but not LTag(WT)-EGFP, engages the cellular PQC pathways leading to its degradation, making it a suitable reporter for the identification of novel PQC components.

**Figure 2:**
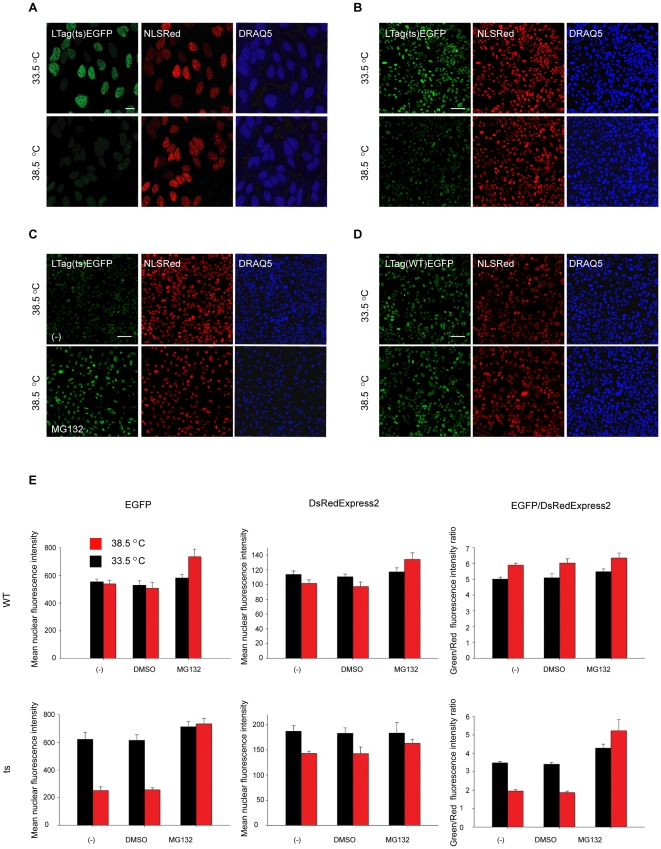
The PQC assay quantitatively measures the degradation of LTag(ts)-EGFP at the restrictive temperature. a) U2OS cells stably expressing LTag(ts)-EGFP and NLS-DsRedExpress2 (NLS-Red) were grown in 384 well-plates for 48 hrs at 33.5°C and then shifted at the indicated temperatures for 24 hrs. DRAQ5 stains the DNA and is used to identify the nuclei. Cells were fixed in paraformaldehyde and imaged sequentially at 488 nm, 568 nm, and 640 nm (40× magnification, 1 imaging field). Scale bar: 20 µm b) Montage of 12 40× fields representing the entire population of cells in a 384-well. Scale bar: 100 µm. c) Same as b), except that cells were treated with MG132 (250 nM). (-) indicates the untreated control. d) Same as b) except that U2OS cells stably expressing the LTag(WT)-EGFP and NLS-DsRedExpress2 were used. e) Histograms representing the quantification of nuclear fluorescence intensities and the EGFP/DsRedExpress2 ratio. Typically >300 cells were imaged per well. Values represent averages +/− S.E.M of 4 experiments.

Having biochemically demonstrated the feasibility of using LTag(ts)-EGFP as a probe to measure PQC in human cells, we tested its usefulness as a reporter in a high-content fluorescence microscopy format. Both LTag(ts)-EGFP and NLS-DsRedExpress2 localized to the nucleus at the permissive temperature (Figure 2A and 2B, 33.5°C). When cells were shifted to the restrictive temperature for 24 h the LTag(ts)-EGFP signal was reduced with no apparent change in the NLS-DsRedExpress2 signal (Figure 2A and 2B, 38.5°C). The qualitative reduction of LTag(ts)-EGFP was confirmed quantitatively. The average LTag(ts)-EGFP intensity was typically reduced by about 66% after 24 h at the restrictive temperature (Figure 2B and 2E), and as a consequence the EGFP/DsRedExpress2 ratio was reduced by ∼50% (Figure 2B and 2E). Single cell analysis confirmed that, upon shift from 33.5°C to 38.5°C, and for the entire population of LTag(ts)-EGFP expressing cells, but not for LTag(WT)-EGFP expressing cells, the distribution of the EGFP/DsRedExpress2 ratio shifted toward lower values (Figure S1C).

The reduction in the LTag(ts)-EGFP signal was due to protein degradation since proteasome inhibition with MG132 at the restrictive temperature resulted in a complete rescue of the mean nuclear green fluorescence intensity and of the EGFP/DsRedExpress2 ratio, with no major alteration in the mean nuclear red fluorescence intensity (Figure 2C and 2E). Furthermore, the observed degradation was specific to the ts-mutant since no loss of EGFP signal was observed when cells expressing LTag(WT)-EGFP and NLS-DsRedExpress2 were shifted to the restrictive temperature (Figure 2D). These results are in line with the biochemical data (Figure 1) and demonstrate that the EGFP/DsRedExpress2 ratio is an accurate and quantitative indicator of PQC activity in the nucleus of human cells.

### A targeted high-throughput RNAi screen for PQC components

**Figure 3:**
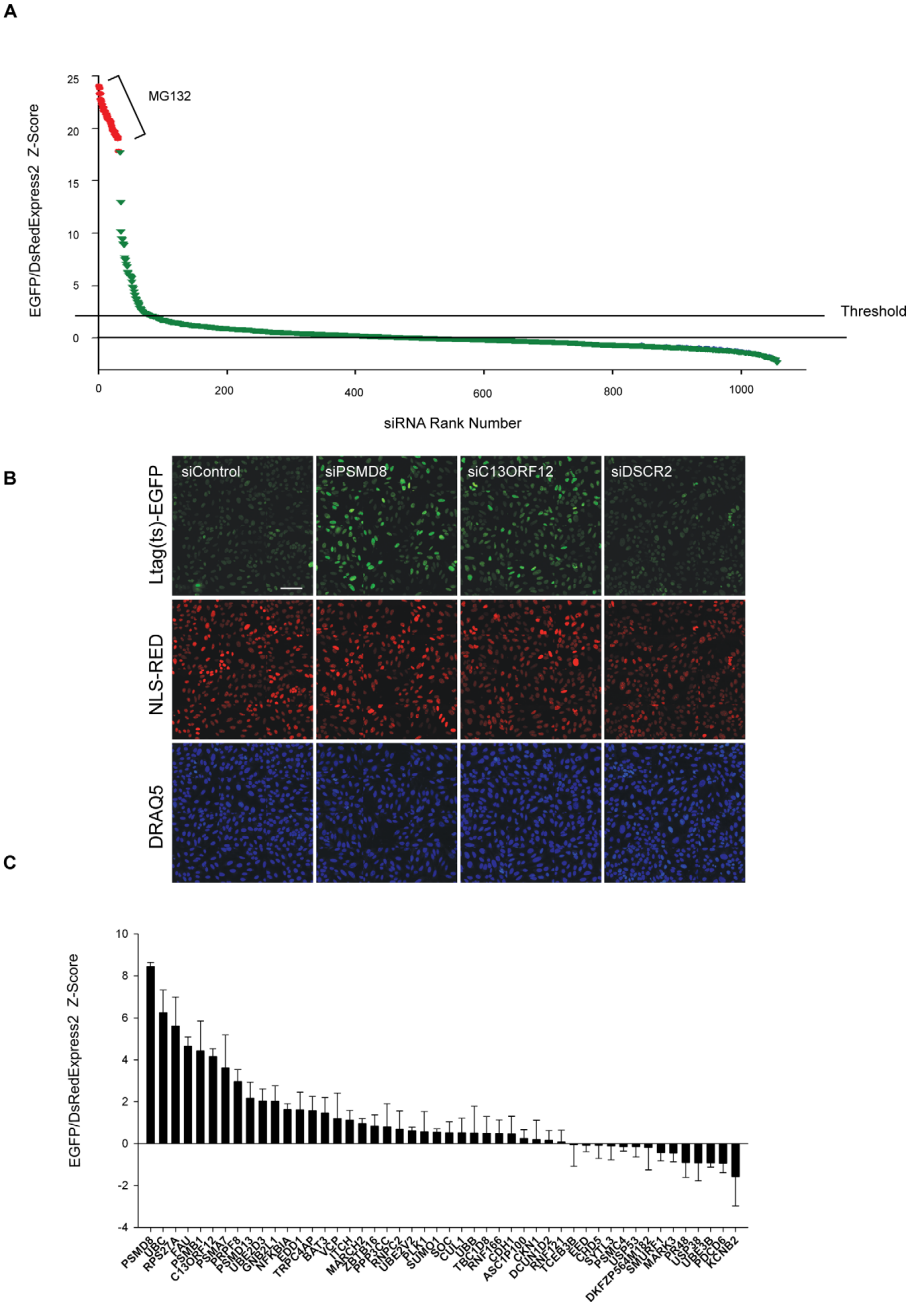
Primary hits from the Ubiquitin Proteasome System (UPS) siRNA screen. a) U2OS cells expressing LTag(ts)-EGFP and NLS-DsRedExpress2 were screened in triplicate at 38.5°C using a library containing 991 siRNA pools targeting human genes belonging to the Ubiquitin Proteasome System (UPS). siRNA pools were ranked according to their Z-score in the EGFP/DsRedExpress2 ratio when compared to the median value of the population. Positive control wells (red) were treated with MG132 (250 nM). The horizontal dashed line represents the threshold (Z = 2) used for the selection of the positive hits. b) U2OS cells expressing LTag(ts)-EGFP and NLS-DsRedExpress2 were treated with an siRNA pool against the *PSMD8* subunit of the 19S proteasome, the 20S proteasome chaperone *C13ORF12*, or the 20S proteasome chaperone *DSCR2/PAC1* for 48 hrs at 33.5°C and then shifted to 38.5°C for 24 hrs. Images shown here are montages of 12 imaging fields at 40× magnification. Scale bar: 100 µm. c) Histogram representing the Z-scores of the EGFP/DsRedExpress2 ratio obtained in the secondary screen at 38.5°C using a validation library containing 48 On-Target Plus siRNA pools targeting selected primary hits from the UPS and Ubi123 screens. U2OS cells expressing LTag(ts)-EGFP and NLS-DsRedExpress2 were used in this experiment. Typically >300 cells were imaged per well. Values represent averages +/− S.E.M of 4 experiments.

In order to identify novel genes of the PQC pathway, we initially used the LTag(ts)-EGFP/NLS-DsRedExpress2 cell line to screen a library containing 991 pools of 4 siRNA oligos targeting human genes involved in the Ubiquitin Proteasome System (UPS) (Table S1). The screen was conducted in triplicate in a 384-well format by reverse-transfecting cells with siRNA pools for 48 h at 33.5°C, followed by a shift to 38.5°C for another 24 h before fixation of cells in paraformaldehyde (See Materials and Methods). We imaged 12 fields per well at 40× magnification in 3 channels in an automated fashion (green, red and far red to detect the DNA stain DRAQ5) for a total of 300–600 cells per RNAi pool. Z′-scores for the EGFP/DsRedExpress2 ratio were calculated using positive (MG132, 250 nM) and negative (non-targeting siRNA pool) controls for each replicate and they ranged from 0.5 to 0.74 (See Materials and Methods). Samples treated with the positive control MG132 had Z-scores ranging from 17.81 to 24.01 (Figure 3A). Of the 991 siRNA pools 57 had a statistically significant effect on the EGFP/DsRedExpress2 ratio corresponding to a hit-rate of 5.7%. Positive hits had EGFP/DsRedExpress2 Z-scores between 2 and 17.72 (Figure 3A and Table S2). Among the hits were 3 out of the 4 genes encoding ubiquitin in the human genome (*UBB*, *UBC* and *RPS27A*), the 20S proteasome chaperone *C13ORF12* (Table S2 and Figure 3B) and 2 genes encoding the ubiquitin-like proteins *FAU* and *SUMO1* (Table S2). In addition, we also identified 25 out of the 33 genes encoding the 26S proteasome core subunits [24], including *PSMD8*, one of the 9 subunits of the 19S proteasome lid (Figure 3B and Table S2). Silencing of *DSCR2*/*PAC1*, another 20S proteasome chaperone involved in the same pathway as *C13ORF12*, did not cause an increase in either LTag(ts)-EGFP fluorescence (Figure 3B) or in the EGFP/DsRedExpress2 ratio (Table S2). These results indicate that the PQC assay is capable of capturing many of the genes whose silencing results in an inhibition of the degradation of misfolded proteins.

Given the prominent role of 26S proteasome and ubiquitin genes in our list of primary hits, we further used the PQC cell line to screen a library containing 600 siRNA pools targeting the expression of all predicted E1-, E2- and E3-ubiquitin ligases in the human genome (Ubi123, Table S3). By using the same criteria (EGFP/DsRedExpress2 Z-score>2), we identified 13 primary hits from this library (Table S4), among them *UBE2D3*, one of the 4 human genes encoding for a protein orthologous to the Ubc4/5 E2 ubiquitin ligase, which was previously shown to participate in the ubiquitination of misfolded proteins in *S. cerevisiae* [25]. As expected, silencing of the proteasome subunits lead to varying cytotoxic effects (Z-scores ranging from 6.08 for *PSMD*1 to 1.34 for *PSMD1*3, equivalent to 33% to 85.3% viability, respectively), as measured by the number of cells present at the end of the experiment, when compared to the median number of cells transfected with the UPS siRNA library (Table S5). On the other hand, under the same conditions silencing of *POMP* had only a minimal effect on cell viability (Z-score of 1.89, equivalent to 79.4% viability) (Table S5).

### Validation of primary hits

To rule out the possibility that the observed biological activity of the siRNA pools was due to non-specific off-target effects, we assembled a validation library of 48 siRNA pools with a modified chemical backbone distinct from that used in the primary screen (See Materials and Methods). This validation library contained most of the primary hits obtained from the UPS and the Ubi123 screens. 5 of the 25 26S proteasome genes were also included as controls (Table S6). Amongst the 48 selected genes (Z-Score>2 in primary screen), 11 also reached a Z-score of >2 when validated against the LTag(ts)-EGFP cell line at 38.5°C. Importantly, 8 out of the top 10 primary screen hits (Z-Score>4) reproduced in this validation experiment (Z-Score>2, Figure 3C, and Table S2 and S4). These results indicate a false positive discovery rate of ∼20%, which is comparable to screens using the same siRNA reagents [26]. We conclude that the identified genes are *bona fide* components of the PQC machinery.

### Identification of constitutive protein degradation components vs. PQC components

**Figure 4:**
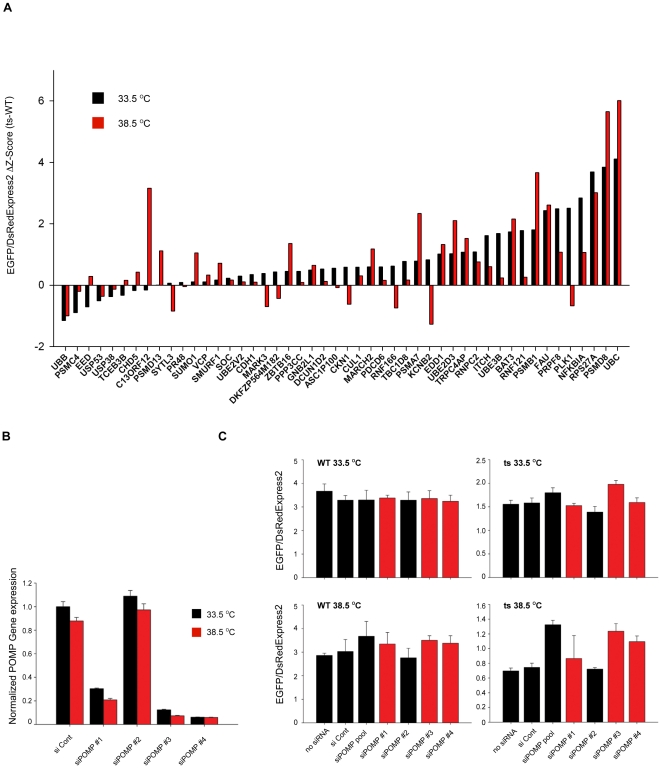
PQC Validation of primary hits. a) U2OS cells expressing either LTag(WT)-EGFP or LTag(ts)-EGFP and NLS-DsRedExpress2 were screened in quadruplicate at 33.5°C or 38.5°C using the validation library described in Figure 3C. The ΔZ-score is calculated as the difference between the EGFP/DsRedExpress2 ratio Z-score obtained using LTag(ts)-EGFP cells and the EGFP/DsRedExpress2 ratio Z-score for LTag(WT)-EGFP expressing cells. *C13ORF12* is also known as *POMP*. b) Deconvolution and measurement by quantitative RT-PCR of the RNA silencing activity of the original siGenome pool of 4 siRNA oligos targeting the *POMP* gene. c) Measurement of the biological activity of the 4 siRNA oligos directed against the *POMP* gene in the PQC activity assay. Red bars represent siRNA oligos possessing siRNA silencing activity (see b)). Values represent averages +/− S.E.M of 4 experiments.

In order to distinguish genes that play a constitutive role in protein degradation from genes specifically involved in PQC of misfolded proteins, we counter-screened cell lines expressing either LTag(WT)-EGFP or LTag(ts)-EGFP at both 33.5°C and 38.5°C. A gene preferentially involved in the degradation of misfolded proteins should show activity in the PQC screen in the cell line expressing the ts and not the WT version of the LTag, and it should do so specifically at the restrictive but not the permissive temperature. By ranking the siRNA pools in the validation library according to their ΔZ-score at either temperature (defined as the difference of the Green/Red Z-score of the ts protein minus the Z-score of the WT protein), we identified siRNA pools that had a high ΔZ-score at both permissive and restrictive temperature. These represent constitutive components of the Ubiquitin-Proteasome System. As expected, siRNA pools targeting genes encoding core members of the protein degradation machinery, such as 26S proteasome subunits (*PSMD8* and *PSMA7*) or ubiquitin (*UBC* and *RPS27A*), had a significant ΔZ-score at both temperatures (Figure 4A). On the other hand, *C13ORF12*, also known as *hUMP1* or *POMP*, showed a high ΔZ-score only at the restrictive but not at the permissive temperature, thus fitting the requirements for a genuine PQC factor (Figure 4A). Furthermore, silencing of *POMP* at 38.5°C did not cause an increase in cytotoxicity when compared to silencing at 33.5°C (94.3% and 87.9% viability, respectively, Table S7). This observation indicates that the differential effect of silencing *POMP* expression on the stability of misfolded proteins at the two temperatures is not artifactually due to an increase in cytotoxicity.

### POMP as a novel target for differential inhibition of proteolytic activity during proteotoxic stress

*POMP* encodes a protein chaperone whose role is to promote the assembly of the 20S proteasome [27]–[30]. In mammalian cells, POMP localizes to both the nucleus and the cytoplasmic face of the ER [31], [32]. In human cells POMP binds to proteasome subunits and facilitates their assembly into the hemi-proteasome, an intermediate in the mature 20S proteasome formation process that is formed by two heptameric rings of α- and β- subunits [24], [28], [29], [33]. Upon completion of this process, two assembled hemi-proteasomes come together to form the mature 20S proteasome and POMP is rapidly degraded in the process [27], [28]. Other proteasome chaperones involved in the same pathway, including *DSCR2/PAC1*, *PAC2*, *PAC3* and *PAC4*, were present in the UPS library used in the primary screen but were not identified as positive hits. The observed biological effect of was not due to off-target activity since three out of four independent siRNA oligos present in the original pool against *POMP* effectively knocked-down *POMP* mRNA transcript levels at both the permissive and at the restrictive temperature (Figure 4B). 2 out of the 3 effective siRNAs against *POMP* showed significant inhibition of LTag-EGFP degradation of the ts allele but not the WT allele and only at the restrictive temperature, confirming its role as a novel component in the proteolytic stress response (Figure 4C). The subcellular localization of POMP was not altered upon heat-shock at 42.0°C in U2OS cells (Figure S1D). Given the partial cytoplasmic localization of POMP in immunofluorescence experiments, we cannot rule out that a small fraction of LTag may be degraded in the cytoplasm in a POMP-mediated manner. POMP was not transcriptionally up-regulated at 38.5°C when compared to 33.5°C in LTag(ts)-EGFP expressing cells (Figure 4B). These results demonstrate a function for POMP in the degradation of a misfolded substrate in the presence of an increased proteolytic load and proteotoxic stress conditions. The finding that, contrary to other genes encoding proteasome subunits such as *PSMD8* or *PSMB1*, silencing of the *POMP* gene specifically inhibited the degradation of LTag(ts)-EGFP at the restrictive temperature, raises the intriguing possibility that POMP activity plays a key role in response to proteotoxic stress.

### Genome-wide siRNA screen for the identification of PQC factors

**Figure 5:**
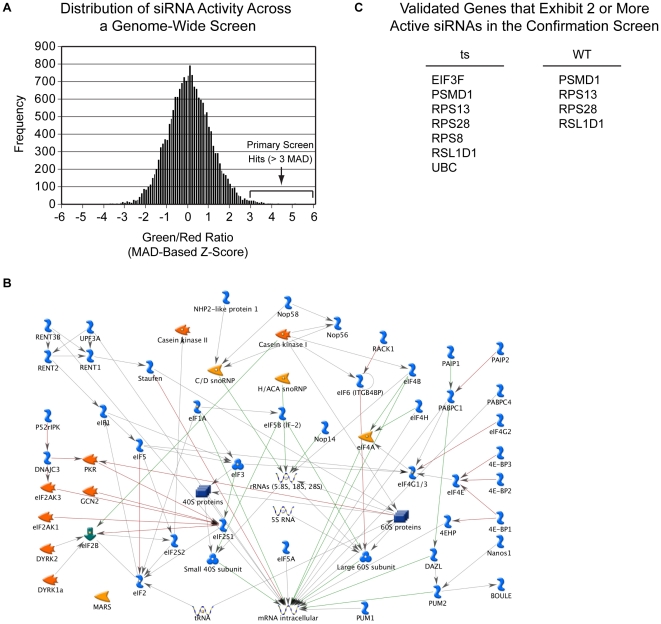
Genome-wide siRNA screen for PQC factors. a) U2OS cells expressing LTag(ts)-EGFP and NLS-DsRedExpress2 were screened against a library containing ∼18, 000 siRNA pools targeting human protein-coding genes. The histogram represents the distribution of the EGFP/DsRedExpress2 ratio robust Z-score measured for each siRNA pool. The location of the EGFP/DsRedExpress2 ratio robust Z-score threshold used to select positive siRNA pools is indicated. b) Analysis of hits from the genome-wide PQC screen reveals significant enrichment for translation/translation initiation processes. Enrichment analysis was conducted using GeneGo Process Networks with a false discovery rate (FDR) of 0.05. 9 of the top 84 genes are found within this network, including EIF3A, EIF3F, NHP2-like protein 1 (NH2L1), NOP56/NOL5A, RPS13, RPS24, RPS28, RPS4X and RPS8. c) U2OS cells expressing LTag(ts)-EGFP or LTag(WT)-EGFP and NLS-DsRedExpress2 were independently transfected for validation with 4 siRNAs targeting 71 of the genes that were scored as positive in the primary PQC screen. Positive hits in this secondary screen were classified as genes whose silencing by at least two independent oligo siRNAs had a EGFP/DsRedExpress2 ratio larger than 140% of the value obtained by transfection of a non-targeting siRNA oligo. The table shows such positive genes in the LTag(ts)-EGFP or LTag(WT)-EGFP expressing cell lines, respectively.

In order to discover additional cellular pathways that affect the stability of misfolded proteins, we performed an unbiased genome-wide RNAi screen. We screened a library of ∼18, 000 pools of 4 siRNAs targeting most of the annotated human genes against the LTag(ts)-EGFP/NLS-DsRedExpress2 cell line at 38.5°C. We identified 84 genes that concomitantly exhibited an EGFP/DsRedExpress2 Z-score of >3, an EGFP Z-score of >1, and viability >60% of the population median (Figure 5A and Table S8), representing a hit rate of ∼0.5%. As previously observed for the UPS-focused siRNA screen, the proteasome subunits genes *PSMD1* and *PSMC4* and the ubiquitin gene *UBC* scored positive, confirming the specificity of the PQC assay. Gene GO Process Network analysis revealed that, when compared to a random sample, the primary hits of the genome-wide PQC siRNA assays were significantly enriched in the category of protein translation (9 out of 84 hits, Fig. 5B). Amongst protein translation factors, we identified 5 subunits of the 40S ribosome (*RPS4X*, *RPS8*, *RPS13*, *RPS24* and *RPS28*), *NOP56/NOL5A*, *NH2PL1*, and 2 subunits of the translation initiation factor EIF3 (*EIF3A* and *EIF3F*; Figure 5B and Table S8) as positive hits.

Hits were validated by independently transfecting the LTag(ts)-EGFP and the LTag(WT)-EGFP cell lines with 4 distinct siRNA oligos against 71 of the genes that scored positive in the primary genome-wide screen (Fig. 5C, Table S9). In the LTag(ts)-EGFP cell line, amongst 284 siRNA tested for validation, 45 had an EGFP/DsRedExpress2 ratio of >140% of the negative control siRNA. We considered genes that were targeted by at least two of these active siRNAs as positive hits. As expected, the gene functions associated with this group were mainly related to the Proteasome (*PSMD1*), Ubiquitin (*UBC*), protein translation initiation (*EIF3F*) and the 40S ribosome (*RPS8*, *RPS13* and *RPS28*) (Figure 5C, Table S9).

### Silencing of the translation initiation factor EIF3F leads to misfolded protein stabilization

As observed when testing the UPS-focused library (Fig. 4A), most validated hits also scored positive in the PQC assay when tested in the LTag(WT)-EGFP/NLS-DsRedExpress2 expressing cell line, indicating that they most likely participate in constitutive pathways of protein degradation (Fig. 5C, Table S9). In contrast, silencing of *EIF3F*, *RPS8* and *UBC* with multiple independent siRNAs showed a preferential increase in the EGFP/DsRedExpress2 ratio in the LTag(ts)-EGFP expressing cell line when compared to the WT counterpart (Figure 5C). This suggests that inhibition of translation leads to the stabilization of misfolded proteins. In support, Western blotting revealed that treatment of LTag(ts) expressing cells with the translation inhibitor cycloheximide led to the stabilization of the temperature-sensitive protein, but not of the WT protein at 38.5°C (Figure S1E). This biochemical assay confirms the observations in the dual fluorescent reporter and shows that inhibition of protein translation *per se* is sufficient to induce the stabilization of a misfolded protein. Altogether, the genome-wide siRNA screen indicates that modulation of translation initiation results in a preferential stabilization of PQC protein substrates when compared to more stable, properly folded proteins. This effect is mediated via the translation initiation complex EIF3.

## Discussion

Here we describe and use a high-content cell-based assay for the discovery of human PQC factors involved in the degradation of misfolded proteins. Fluorescent reporters for generic proteasome-mediated protein degradation have been a useful tool to study the activity of these pathways in cell culture [34], however, no assay specifically dedicated to the discovery of mammalian PQC has been available. Our approach to measure PQC activity in human cells involves fusing EGFP to a temperature-sensitive version of the SV40 virus Large T-antigen and measuring its degradation in intact cells. We find that the temperature-sensitive SV40-LTag reporter protein is efficiently degraded upon switching to the restrictive temperature but is stable at the permissive temperature. Degradation is not due to defective folding of EGFP at 38.5°C because an identical fusion with the wild-type, stable version of the substrate is not affected at this temperature. In contrast to other temperature-sensitive proteins that have been used to measure the activity of the PQC pathway in *S.cerevisiae*, and which are generally short-lived proteins [12], [35], [36], the LTag(ts) has a long half-live in vivo [37] with ∼50% of LTag(ts) degraded within 12 hours after switch to the non-permissive temperature (Figure S1A). As such, our assay is well suited to probe the still poorly understood fate of long-lived proteins that are often of particular relevance for disease. Prominent examples include the nuclear lamins, which accumulate at the nuclear periphery in the premature aging disorder Hutchinson-Gilford Progeria Syndrome, or huntingtin, a protein that can pathologically misfold in the nucleus [38]. A further advantage of our assay compared to previous reporters of protein degradation [34], is its ability to discriminate effects that are unrelated to protein degradation based on monitoring levels of the internal control NLS-DsRedExpress2. Measuring the EGFP/DsRedExpress2 fluorescence ratio is a quantitative, reproducible and robust way to measure the PQC activity in human cells.

By directly comparing the effects of the same siRNA treatment on LTag(ts)-EGFP and LTag(WT)-EGFP, we were able to distinguish genes that are generally involved in protein degradation, such as the ones encoding for proteasome subunits, from ones that are preferentially involved in the acute response to LTag(ts) misfolding at the restrictive temperature, such as the proteasome chaperone POMP or the translation initiation factor EIF3F.

The results of an initial focused siRNA screen of ∼1600 factors involved in UPS strongly indicate that this assay system is capable of capturing many of the factors that are essential for PQC. By using the EGFP/DsRedExpress2 fluorescence ratio as a readout, we identified 28 out of the 33 genes that encode for 26S proteasome subunits among a total of 78 hits in our primary screen, thus validating the rationale behind the design of the PQC assay and demonstrating a low false negative discovery rate. In addition, from a biological standpoint, the results of the PQC screen described here clearly indicate that genes encoding 26S proteasome subunits, ubiquitin, the 20S proteasome chaperone POMP, and the E2 ubiquitin ligase UBE2D3, are the only ones whose individual loss is sufficient to significantly inhibit degradation of the misfolded temperature sensitive protein. We cannot entirely rule out the possibility that other such genes exist and that they are not detected here due to incomplete siRNA gene silencing, but we consider it unlikely given our high success rate in identifying 26S proteasome subunits in our screen. The absence of many “single hits” among known 26S proteasome-associated proteins indicates a high level of redundancy among cellular pathways involved in the degradation of misfolded proteins. In the future it will be interesting to test selected pair-wise combinations of siRNA pools targeting independent genes to uncover genetic compensatory interactions between pathways involved in PQC.

An unbiased genome-wide screen confirmed and extended the findings obtained in the focused UPS screen. siRNA silencing of the proteasome gene *PSMD1*, of the ubiquitin gene *UBC* and of genes encoding subunits of the EIF3 translation initiation factor and of the 40S Ribosome lead to the stabilization of the LTag(ts) at 38.5°C. By silencing the expression of one gene at a time, the results of the genome-wide PQC screen thus suggests that in human cells, beside cellular activities related to protein degradation, protein translation initiation is the other main non-redundant pathway which, when inhibited, regulates the stability of unstable or misfolded proteins. This is in line with previous findings in lower eukaryotes, obtained by genome-wide siRNA screens for modifiers of protein aggregation [19], [21]. We find that the genome-wide siRNA screen had a significantly lower validation rate when compared to the UPS focused screen. This might be due to the fact that the UPS library is pre-enriched in target genes related to protein degradation thus increasing the hit rate. In addition, due to the size of the genome-wide library, for logistical reasons the genome-wide screen was run once as opposed to three times for the smaller UPS screen. Furthermore, while validation of the genome-wide screen was done using pools of RNAi oligonucleotides of distinct chemistry from those used in the primary screen, validation of the small scale screen was performed by deconvolving the RNAi pools used in the in the primary screening. Finally, compared to the UPS screen, the degree of saturation was lower in the genome-wide screen allowing for the possibility of affecting hit accuracy. Given the relatively strong predominance of protein degradation and translation components amongst the positive hits, it is likely that these additional factors would fall into these functional classes as well.

In contrast to most identified factors, the 20S proteasome chaperone POMP and the EIF3F subunit of the EIF3 translation initiation complex had a differential effect on the stability of LTag(ts)-EGFP. POMP was the first protein shown to assist in the formation of the mature 20S proteasome [27], and it is considered an essential factor during assembly of the 20S proteasome [30], [33]. As such it is not surprising that it was identified in our primary screen at 38.5°C as a PQC component. Contrary to our expectations, however, in contrast to other 26S proteasome subunits, silencing of *POMP* did not have any significant effect at 33.5°C. One possible explanation for this result is that increased POMP activity is preferentially necessary in the presence of an increased load of misfolded proteins, such as at 38.5°C. This is consistent with the increased expression of *POMP* at the mRNA level during proteotoxic chemical treatments, such as in response to MG132 or IFN- γ [29], [30], [39]. Alternatively, siRNA silencing of *POMP* might only partially lower proteasome activity to levels that are sufficient for the clearance of misfolded LTag(ts)-EGFP at the permissive temperature, but these become limiting once the temperature is increased and the pool of misfolded proteins increases in quantity. In either case, these results identify *POMP* as a “single-hit” target for the specific inhibition of the clearance of misfolded proteins during conditions of enhanced proteotoxic stress. This finding is possibly of relevance to cancer treatment since certain types of tumours experience increased proteotoxic stress [40]. Hence, the PQC assay described here might be a useful tool to identify alternative targets for pharmacological inhibition of PQC activities, beside the already known effect of 26S proteasome and HSP90 inhibitors in the context of cancer treatment [41], [42].

EIF3 is a multi-subunit protein complex that acts as platform to bring together the 40S ribosome and several other translation initiation factors to promote translation initiation [43]. The identification of a component of the EIF3 complex as a specific factor for degradation of misfolded proteins when compared to the degradation of more stable substrates raises the intriguing possibility that in mammalian cells the EIF3 translation initiation complex might be important for coupling *de novo* protein synthesis with a quality control step that eliminates misfolded polypeptides. In support of a functional and physical link between EIF3 and PQC, biochemical fractionation experiments in S.*pombe* revealed the existence of a protein supercomplex containing ribosomes, proteasomes, chaperones, EIF3 and other components of the translation machinery [44]. Furthermore, in the same organism, the eIF3e subunit is necessary for the nuclear localization of the proteasome [44]. Possible interactions between the protein translation and degradation machinery are also suggested by the fact that the translation elongation factor EIF1A binds the proteasome and promotes the destruction of newly synthetized ubiquitinated proteins [45], [46]. In addition, it is remarkable that an estimated 30% of all the newly synthetized polypeptides are co-translationally degraded in vivo, suggesting tight coupling of these processes [47], [48]. Alternatively, translation inhibition in mammalian cells might lead to ubiquitin depletion and block proteasome mediated protein degradation, as previously observed in S.*cerevisiae* [49]. However, this is unlikely since ubiquitin has a significantly longer half-life in mammalian cells and extended treatment of cells with cycloheximide does not negatively affect proteasome function [50]–[52]. Regardless of the details of the interplay between translation inhibition, ubiquitin levels and PQC, the identification of several translational components made possible by use of an unbiased genome-wide screen in mammalian cells provides a first hint for such coupling and lays the foundation for full elucidation of these events in higher eukaryotes.

## Materials and Methods

### Cloning

pcDNA3-LTag(WT) was generated by cloning a PCR BamHI/EcoRI fragment obtained from pBABE-neo LargeTc (Addgene plasmid #1780) into pcDNA3 (Invitrogen) cut with the same enzymes. pcDNA3-LTag(ts) expressing the tsa209 allele of LTag [22] carrying a P427L mutation was created with the Quickchange site-directed mutagenesis kit (Stratagene) using pcDNA3-LTag(WT) as a template. The primers used were FW: CTGGCTGTTTAAAGGACTGATTGATAGTGGTAAAACTACATTAGCAGCTGC, and RW:GCAGCTGCTAATGTAGTTTTACCACTATCAATCAGTCCTTTAAACAGCCAG. Retroviral vectors pQXCIN-LTag(WT) and pQXCIN-LTag(ts) were generated by cloning a BamHI/BamHI fragment from pcDNA3-LTag(WT) and pcDNA3-LTag(ts), respectively, into the bicistronic vector pQCXIN (Clontech) cut with the same enzymes. pQXCIN-LTag(WT)-EGFP and pQXCIN-LTag(ts)-EGFP were generated in two rounds of PCR using either pQXCIN-LTag(WT) or pQXCIN-LTag(ts), respectively, and pEGFPN1-HMGB1 [53] as templates. In the first round, an N-terminal fragment containing LTag ORF with primers FW: GGCGGCGGATCCCGCCACCATGGATAAA, RW: AGAGCCTCCGCCTCCAGAGCCTCCGCCTCCTGATGTTTCAGGTTCAGGGG and a C-terminal fragment containing EGFP ORF with primers FW: GGAGGCGGAGGCTCTGGAGGCGGAGGCTCTGTGAGCAAGGGCGAGGAGCTG, RW: GGAGGCGGAGGCTCTGGAGGCGGAGGCTCTGTGAGCAAGGGCGAGGAGCTG. The PCR fragments were gel purified, denatured, annealed and used in a second round of PCR using the external primers. The products from the second round of PCR were digested with BamHI/EcoRI, gel purified and cloned into pQCXIN cut with the same enzymes. To generate pQXCIN-LTag(WT)-EGFP-IRES-NLSDsRedExpress2 and pQXCIN-LTag(ts)-EGFP-IRES-NLS-DsRedExpress2, a BstI/XhoI PCR fragment containing DsRedExpress2 N-terminally tagged with the nuclear localization signal from LTag was amplified from pLVXDsRedExpress2 (A kind gift of B. Glick, [54] using primers FW: CGGCCCACAACCATGGCTCCAAAGAAGAAGAGAAAGGTCGATAGCACTGAGAACGTCATC, RW: GGCGGCCTCGAGCTACTGGAACAGGTGGTGGC. The PCR product was cut with BstI/XhoI and cloned into either pQXCIN-LTag(WT)-EGFP or pQXCIN-LTag(ts)-EGFP cut with the same enzymes.

### Cell Culture

U2OS (human osteosarcoma) cells were obtained from ATCC. Unless otherwise specified, cells were grown in McCoy's 5A Medium (Invitrogen), 15% Fetal Bovine Serum, 100 U/ml penicillin and 100 µg/ml streptomycin at 33.5°C in 5% CO_2_. Polyclonal cell lines expressing the different LTag constructs were generated by retroviral infection of U2OS cells as previously described ([55]). Cells stably expressing untagged alleles of LTag were selected in 500 µg/ml G418, whereas a polyclonal population of U2OS cells concomitantly expressing LTag-EGFP (Either WT or ts) and NLS-DsRedExpress2 were selected by FACS. In order to trigger the degradation of the temperature sensitive protein, cells were switched from 33.5°C to 38.5°C for 24 hrs. Except where otherwise specified, MG132 (Calbiochem) was used at a final concentration of 250 nM for 24 hrs.

### siRNA reagents

siGenome pools and OnTargetPlus pools of 4 siRNA oligos per gene were purchased from Dharmacon. Cells were transfected using Dharmafect1 (ThermoFisher Scientific). The four independent oligos present in the original siGenome pool targeting hUMP1 were ordered from Dharmacon. The Ubiquitin Proteasome (UPS) library contains 991 genes involved in proteasome and ubiquitin metabolism (Table S1). The Ubiquitin sets 1, 2 and 3 containing genes encoding for E1, E2 and E3 ubiquitin ligases were purchased from Dharmacon (Table S3). Genes selected for the secondary screens and the relative OnTargetPlus siRNA oligos sequences are described in Table S5.

### Western Blotting and antibodies

Total cell lysates were obtained by direct lysis of cells in 1× Laemmli Buffer [56]. Cell lysates were boiled at 95°C, run on a 4–12% SDS-PAGE Bi-Tris NUPAGE gel (Invitrogen), transferred to a PVDF membrane (Millipore) and probed with appropriate primary antibodies and HRP-labelled secondary antibodies. The Western blots were developed using an ECL kit from Amersham/Pharmacia. The following antibodies were used: mouse monoclonal anti-LTag (BD Pharmingen), mouse monoclonal anti-β-Tubulin (Sigma), goat polyclonal anti-POMP (Protoassemblin, St. Cruz, E-20).

### POMP localization upon heat shock

U2OS cells were grown at 37.0°C in McCoy's 5A Medium, 15% Fetal Bovine Serum, 100 U/ml penicillin and 100 µg/ml streptomycin. 10 mM Hepes pH 7.4 was added to the medium and cells were further incubated for 2 hrs at 42.0°C before fixation in 4% paraformaldehyde in PBS. Indirect immunofluorescence microscopy using goat polyclonal anti-POMP (Protoassemblin, St. Cruz, E-20) was performed as previously described [57].

### qRT PCR

qRT PCR was performed as previously described [57]. Primers used for POMP real time PCR were FW: TTTGCCTAGTCATCCCCTTG, RW: CGGAGCAAATAGACCCTGAA. Primers used for PSMB5 real time PCR were FW: TTTGCCTAGTCATCCCCTTG, RW: CGGAGCAAATAGACCCTGAA.

### siRNA transfection of cells in a 384-well format

Cells were transfected in triplicate on independent days with siRNA oligos at a final concentration of 50 nM in a reverse format using a Janus automated liquid handler (Perkin-Elmer). First, 3.75 µl of OPTIMEM (Invitrogen) were transferred into each well of an empty PE-Cell Carrier 384-well imaging plate (Perkin-Elmer). Then, 1.25 µl of a 1 µM siRNA stock in OPTIMEM were added to the. Finally, 5 µL of diluted Dharmafect1 were added onto the siRNA. Plates were incubated 20 min at RT and then 4000 cells/well were seeded in the plate in a 15 µl volume of McCoy's 5A Medium, 15% Fetal Bovine Serum, 100 U/ml penicillin and 100 µg/ml streptomycin using a Multidrop Combi automated dispenser (ThermoFisher Scientific). Cells were incubated at 33.5°C for 48 hrs and then switched to 38.5°C for another 24 hrs, or for a control plate, left at 33.5°C for the entire duration of the experiment.

### Automated imaging

All the fixation, washing and staining steps were performed using a Biotek EL406 automated plate washer. Cells were first fixed by adding 25 µl/well of 4% paraformaldehyde in PBS directly into the culture medium and incubated for 5 min at RT. Cells were then washed 3× in PBS and stained with DRAQ5 (Biostatus Limited) 1∶5000 in PBS. The automated imaging steps were performed using an Opera system (Perkin Elmer). 12 confocal 40× imaging fields were sequentially imaged using a 488/640 nm excitation laser (1^st^ acquisition) and then using a 568 nm excitation laser (2^nd^ acquisition). Images were analysed using the Acapella software package (Perkin-Elmer). The Green/Red ratio was calculated as the ratio between the average nuclear intensity signal per well in the 488 nm channel and the average nuclear signal per well at the 568 nm channel. Typically >300 cells were imaged per well.

### Statistical analysis

Normalized Z-score values for the Green/Red ratio and the siRNA rank were calculated using the CellHTS2 package [58]. siRNA pools with Z-Score>2 were then selected for secondary validation.

### Genome-wide Screening

Genome-wide screening was conducted using Dharmacon's siGENOME SMARTPool library (ThermoFisher Scientific). This library targets ≈18,000 human genes with pools of 4 siRNAs targeting each gene. Briefly, siRNA (0.8 pmol) was spotted to 384 well plates wells (Corning 3712) using a VPrep liquid handler (Velocity11, Agilent Technologies). Lipofectamine RNAiMax (0.1 µl, Invitrogen) was then added in 20 µl of serum free media (McCoy's 5A). Transfection reagent and siRNA were complexed for 45 minutes at RT before adding cells (1,500) to 20 µl of mixtures comprising 20 nM siRNA in media containing 15% serum. Cells were incubated for 48 h at 33.5°C before switching to 38.5°C for an additional 24 h. Cells were then fixed by adding 40 µl of 4% paraformaldehyde in PBS and incubating for 10 minutes at rt. Cells were washed with PBS (40 µl×2) and stained with Hoechst 33342 (1 µg/ml).

Cells were imaged on an ImageXpress Micro automated microscope (Molecular Devices). Images were acquired using a 4× objective and appropriate exposure times for each wavelength. Analysis was performed using MetaXpress software (version 3.1.0.65, Molecular Devices). For each well, the average normalized intensities for both green and red wavelengths (derived using the median green or red intensity value for cells transfected with Dharmacon non-targeting negative control siRNA #5) were used to calculate green/red ratios.

## Supporting Information

Figure S1 a) U2OS cells stably expressing either a wild-type (WT) or a temperature-sensitive (ts) allele of LTag were grown at 33.5°C for 48 hrs and then shifted to 38.5°C for the indicated amount of time. Total cell lysates were probed in Western Blotting with the indicated antibodies. b) Same as a), except that cells were either treated with DMSO or the proteasome inhibitor MG132 at a final concentration of 2 µM. c) Distribution of the single-cell EGFP/DsRedExpress2 ratios in populations of U2OS cells expressing either LTag(WT)-EGFP or LTag(ts)-EGFP and NLS-DsRedExpress2. d) Indirect immunofluorescence images of U2OS cells grown for two hours at 37.0°C (Control) or 42.0°C (Heat-Shock), fixed in paraformaldehyde and stained with an antibody against POMP. DAPI, 4′, 6′-diamidino-2-phenylindole. Scale bar: 20 µm. U2OS cells stably expressing either a wild-type (WT) or a temperature-sensitive (ts) allele of LTag were grown at 33.5°C for 48 hrs and then shifted to the indicated temperature for the indicated amount of time. e) Same as a), except that cells were treated either with DMSO or with the protein translation inhibitor cycloheximide (50 µg/ml) for 24 hrs. Protein lysates of treated cells were probed in Western Blotting with the indicated antibodies.
(TIF)

Table S1 Sequences of the siGenome 991 siRNA pools contained in the UPS library.
(XLSX)

Table S2 Results of the PQC screen at 38.5°C using the siGenome UPS library and cells expressing LTag(ts)-EGFP and NLS-DsRedExpress2. Z-scores for the EGFP/DsRedExpress2 ratio are shown. Pos: Positive control samples treated with MG132 (250 nM). siRNA pools highlighted in blue represent positive hits in the screen.
(XLSX)

Table S3 Sequences of the 600 siGenome siRNA pools contained in the Ubiquitin E1, E2 and E3 ligases library (Ubi123).
(XLSX)

Table S4 Results of the PQC screen at 38.5°C using the siGenome Ubi123 library and cells expressing LTag(ts)-EGFP and NLS-DsRedExpress2. Z-scores for the EGFP/DsRedExpress2 ratio are shown. Pos: Positive control samples treated with MG132 250 nM. siRNA pools highlighted in blue represent positive hits in the screen.
(XLSX)

Table S5 Cell viability data for the UPS and the Ubi 123 screens using cells expressing LTag(ts)-EGFP and NLS-DsRedExpress2 at 38.5°C, as measured by counts of nuclei present at the end of the experiment.
(XLSX)

Table S6 Sequences of the 48 On-Target Plus siRNA pools contained in the validation library.
(XLSX)

Table S7 Cell viability data for the secondary validation screens using cells expressing LTag(ts)-EGFP and NLS-DsRedExpress2 at 33.5°C and 38.5°C, as measured by counts of nuclei present at the end of the experiment.
(XLSX)

Table S8 List of positive hits (EGFP/DsRed Z-score>3, EGFP Z-score>1) from the genome-wide PQC screen in LTag(ts)-EGFP and NLS-DsRedExpress2 expressing cells at 38.5°C. Full results of the genome-wide PQC screen are also shown.
(XLSX)

Table S9 Results of the validation screen in either LTag(ts)-EGFP or LTag(WT)-EGFP and NLS-DsRedExpress2 expressing cells at 38.5°C for 71 of the positive hits from the primary genome-wide screen. siRNA pools highlighted in blue represent positive hits in the primary screen. Positive genes were defined as those for which silencing by at least 2 siRNA oligos resulted in a EGFP/DsRed ratio of >140% of the negative siRNA control. A conversion table that reflects the different gene symbol nomenclature adopted by the siRNA providers for the primary and validation screen (Dharmacon and Qiagen, respectively) is also provided. The asterisk indicates that *SERPINA13* is a pseudogene. For this reason *SERPINA13* was not included in the final table shown in Figure 5C).
(XLSX)
