# Supplementary figures and images for: Identification of Mammalian Protein Quality Control Factors by High-Throughput Cellular Imaging
Source: PLoS One. 2012 Feb 20;7(2):e31684. doi: 10.1371/journal.pone.0031684 (PMC3282772; doi:10.1371/journal.pone.0031684)

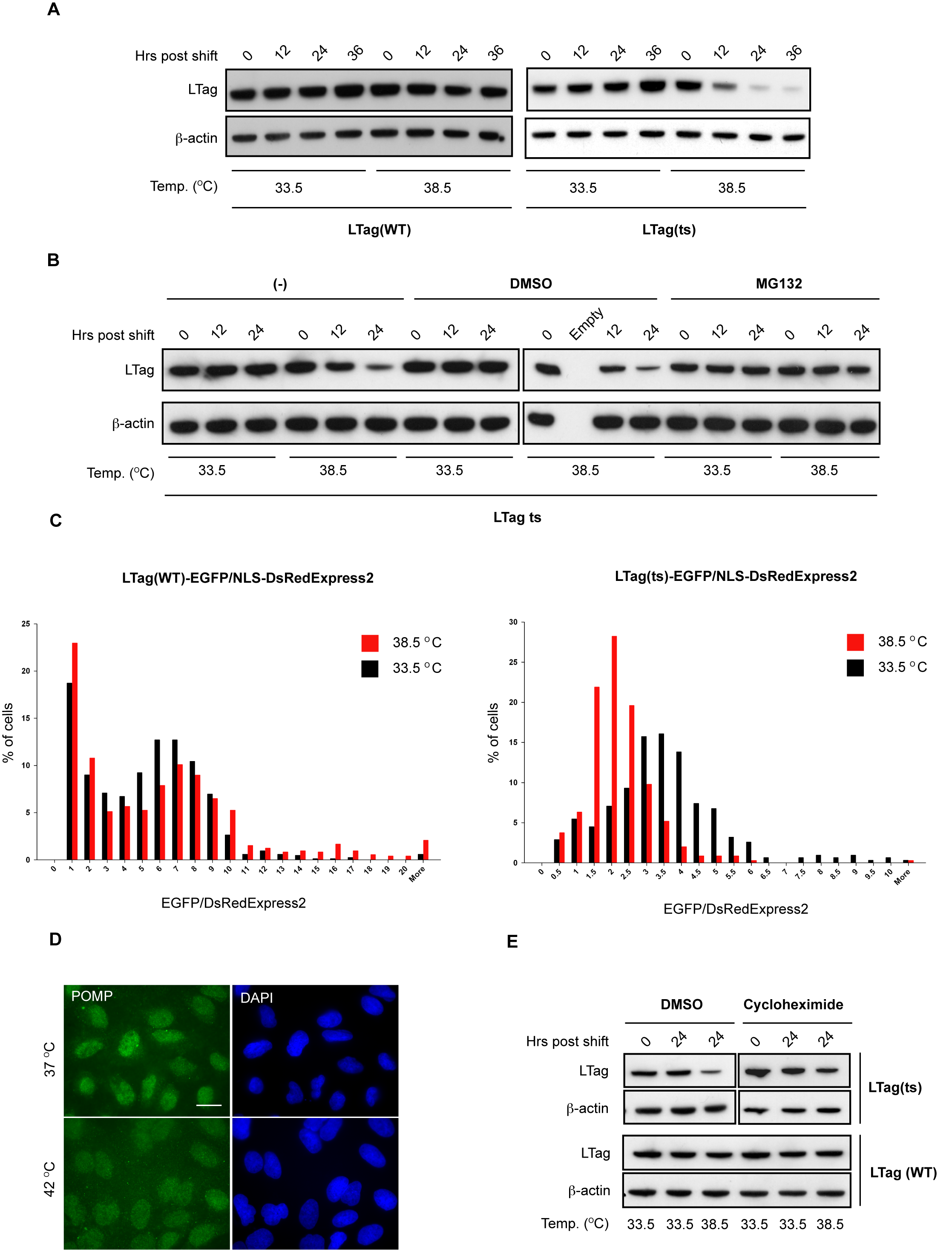

Supplement: Figure S1 — a) U2OS cells stably expressing either a wild-type (WT) or a temperature-sensitive (ts) allele of LTag were grown at 33.5°C for 48 hrs and then shifted to 38.5°C for the indicated amount of time. Total cell lysates were probed in Western Blotting with the indicated antibodies. b) Same as a), except that cells were either treated with DMSO or the proteasome inhibitor MG132 at a final concentration of 2 µM. c) Distribution of the single-cell EGFP/DsRedExpress2 ratios in populations of U2OS cells expressing either LTag(WT)-EGFP or LTag(ts)-EGFP and NLS-DsRedExpress2. d) Indirect immunofluorescence images of U2OS cells grown for two hours at 37.0°C (Control) or 42.0°C (Heat-Shock), fixed in paraformaldehyde and stained with an antibody against POMP. DAPI, 4′, 6′-diamidino-2-phenylindole. Scale bar: 20 µm. U2OS cells stably expressing either a wild-type (WT) or a temperature-sensitive (ts) allele of LTag were grown at 33.5°C for 48 hrs and then shifted to the indicated temperature for the indicated amount of time. e) Same as a), except that cells were treated either with DMSO or with the protein translation inhibitor cycloheximide (50 µg/ml) for 24 hrs. Protein lysates of treated cells were probed in Western Blotting with the indicated antibodies. (TIF) [file pone.0031684.s001.tif]
